# Metabolomic Analysis of Tomato Plants Treated With Garlic Extract, *Bacillus subtilis* , and Their Combination for Defense Against Bacterial Wilt

**DOI:** 10.1002/pei3.70102

**Published:** 2025-12-10

**Authors:** Sinhle Madlhophe, Udoka Vitus Ogugua, Fikile Nelly Makhubu, Ntakadzeni Edwin Madala, Sandiswa Figlan

**Affiliations:** ^1^ Department of Agriculture and Animal Health, Science Campus University of South Africa Florida South Africa; ^2^ Laboratories and Horticulture Centre, Science Campus University of South Africa Florida South Africa; ^3^ Department of Biochemistry University of Venda Thohoyandou South Africa

**Keywords:** biocontrol, caffeoyl glucaric acid, chlorogenic acid, metabolomics, physiological responses, plant defense, *Ralstonia solanacearum*

## Abstract

*Ralstonia solanacearum* , the causal agent of bacterial wilt, severely disrupts the vascular function of tomato plants, leading to significant yield losses. This study aimed to investigate the metabolomic shifts in tomato plants treated with garlic ( *Allium sativum* ) crude extract, *Bacillus subtilis* , and their combination, to assess their roles in enhancing resistance to *R. solanacearum* . Metabolomic profiling was conducted using ultra‐high performance liquid chromatography coupled with quadruple time‐of‐flight mass spectrometry (UHPLC‐qTOF‐MS) to identify and quantify key metabolites associated with stress response. Multivariate statistical analysis (MVDA) tools, viz. principal component analysis (PCA) and the orthogonal projection to latent structures‐discriminant analysis (OPLS‐DA) loading scatter plot were used to identify the metabolites that are positively and negatively correlated to bacterial wilt infection. The profiling revealed that garlic extract up‐regulated key phenolic compounds, including chlorogenic acid and caffeoyl glucaric acid, which contribute to pathogen defense by reinforcing cell structures and mitigating oxidative stress. Chlorogenic acid accumulation was notably prominent in garlic‐treated plants, while caffeoyl glucaric acid exhibited variable regulation across the treatments. Flavonoid levels were generally down‐regulated, indicating a metabolic shift favoring phenylpropanoid pathways in response to disease stress. Additionally, lipid‐related metabolites, such as 12‐dienoate, were reduced in the combined treatment, whereas Juniperoside III was up‐regulated in *B. subtilis* ‐treated plants, suggesting selective regulation of saponin metabolism. These findings indicate that garlic extract enhances plant defense primarily through phenylpropanoid‐mediated structural reinforcement, while *B. subtilis* contributes to disease suppression through microbial interactions rather than significant metabolic shifts. Understanding these metabolic trade‐offs offers valuable insights into optimizing bacterial wilt management strategies, ultimately improving tomato resilience and productivity.

## 1 Introduction

Tomato ( *Solanum lycopersicum* L.), a staple crop with significant economic and nutritional value, faces widespread challenges due to bacterial wilt, a devastating disease caused by *Ralstonia solanacearum* (Vats et al. 2022; Phiri et al. 2024). This pathogen invades the vascular tissues, resulting in rapid wilting, growth inhibition, and eventual plant death, substantially impacting tomato yields worldwide (Kunwar et al. 2020; Oussou et al. 2020). Conventional control methods for bacterial wilt, including chemical treatments, often fall short due to the pathogen's persistence and adaptability in the soil environment (Nihorimbere et al. 2024). As a result, there is increasing interest in sustainable alternatives such as biological control agents and plant‐derived compounds. However, a deeper understanding of the biochemical mechanisms underlying tomato's response to *R. solanacearum* and biocontrol treatments is essential for optimizing these strategies.

Metabolomics provides a valuable tool for addressing this gap by offering a systems level perspective on metabolic changes in response to biotic stress (Ghatak et al. 2018; Allwood et al. 2021). Through advanced analytical tools such as liquid chromatography mass spectrometry (LC–MS), thousands of metabolites can be detected, catalogued, and compared, providing insight into the underlying biochemical and physiological responses of plants to pathogen invasion and biocontrol treatments (Klassen et al. 2017; Wishart 2019). Unlike other omics approaches, metabolomics reflects the end products of gene expression and provides a snapshot of the plant's metabolic response to stress. This is particularly valuable in assessing plant‐pathogen interactions, as metabolite changes often correspond to immediate defense responses (Maroli et al. 2018). By analyzing these metabolic changes, metabolomics can identify biomarkers and metabolic pathways central to plant defense, offering targets for breeding or further enhancement of biocontrol treatments (Adeniji and Babalola 2020). Metabolites such as phenolic compounds, flavonoids, and alkaloids have been associated with improved defense responses in plants (Aoun 2017; Appu et al. 2021; Sood et al. 2021; Jha and Mohamed 2022). Furthermore, metabolomics can reveal how these biocontrol agents influence plant growth pathways, supporting both disease resistance and overall plant health (Adeniji and Babalola 2020; Salem et al. 2020; Lewis et al. 2022).

Among the promising agents in biological control of bacterial wilt in tomato are *B. subtilis* and garlic crude extract. Both agents have demonstrated potential in enhancing tomato resilience to bacterial wilt (Gaurav 2022; Kumar et al. 2024). *Bacillus subtilis* is known for its ability to induce systemic resistance in plants, activating pathways that strengthen cell walls and produce antimicrobial compounds, thereby equipping the plant to better resist pathogenic attacks (Zhou et al. 2021). While garlic extract, on the other hand, is rich in bioactive compounds including allicin, which is known for its antimicrobial properties. Studies have shown that garlic extract can effectively inhibit the growth of *R. solanacearum* , reducing bacterial infection and severity in tomato plants (Wamani 2020). Mougou and Boughalleb‐M'hamdi (2018) have clearly demonstrated that a combined application of biological control agents such as garlic extract and *Pseudomonas fluorescens* can enhance synergistic effects, strengthening resistance mechanisms and offering broader protection against pathogens.

By comparing the metabolic profiles of treated and untreated tomato plants, this study aims to identify key metabolites and pathways responsible for the enhanced resistance against bacterial wilt when treated with garlic extract, *B. subtilis* , and the combination of these two treatments. It was hypothesized that tomato plants treated with garlic crude extract and *B. subtilis* , either individually or in combination, will exhibit significant alteration in the metabolomic profiles in response to bacterial wilt. The findings of the study have the potential to deepen our understanding of tomato defense mechanisms, contribute to more effective disease management strategies, and facilitate the identification of metabolic markers for resistance breeding.

## 2 Materials and Methods

### 2.1 Description of Study Site and Plant Materials Used

The experiment was conducted at the University of South Africa (UNISA), Florida Campus in a greenhouse environment. The facility is located at latitude S26° 9.501′ and longitude E27° 54.113′ in Johannesburg, Gauteng Province, South Africa. The temperature within the greenhouse was maintained at a range between 28°C and 30°C using an automated climate control system, with a 12‐h light–dark cycle to simulate natural day and night conditions. To regulate internal temperature and humidity, the greenhouse was equipped with essential climate control components, including a fan and wet walls. The fan facilitated air circulation, preventing heat buildup. The wet wall system, consisting of a water‐permeated pad, worked in conjunction with the fan to cool the internal environment through evaporative cooling.

Disease‐free seeds of the susceptible tomato cultivar “Heinz 1925” (Kim et al. 2018) were purchased from the reputable seed supplier, Starke Ayres (Johannesburg, South Africa). The seeds were planted on clean and disease‐free planting trays. Two seeds were planted in each hole/cavity of the planting tray. Thinning‐out was done where more than one seed germinated to lessen competition and promote healthy seedlings for the experiment. Once the tomato seedlings developed a set of true leaves, they were fertilized weekly with Nitrosol Organiksol liquid fertilizer (5 mL/L of water) following the method adapted from Bergna et al. (2018). This fertilizer provides essential plant nutrients, including 60 g/kg nitrogen (N), 20 g/kg phosphorus (P), 58 g/kg potassium (K), 7 g/kg magnesium (Mg), 6 g/kg calcium (Ca), and 4 g/kg sulfur (S). It also contains essential micronutrients such as 60 mg/kg iron (Fe), 1 mg/kg copper (Cu), 1 mg/kg zinc (Zn), 40 mg/kg manganese (Mn), 23 mg/kg boron (B), 15 mg/kg molybdenum (Mo), and gibberellic acid (0.003 g/kg). Seedlings were also monitored to ensure adequate hydration, and watering was done manually when needed.

Six weeks after planting, each seedling that had developed a set of true leaves was transplanted into a 21 cm pot, providing enough space for root development. The potting soil (Garden Master) used for this experiment was sourced from Builders Warehouse, a reputable commercial supplier in Florida, Johannesburg. This high‐quality soil mix is designed to provide the necessary nutrients and proper drainage, creating an ideal growing medium for the tomato plants throughout the experimental period. During transplanting, only healthy seedlings were selected, ensuring uniformity in height, no pest damage, and no signs of nutritional deficiencies or diseases. This careful selection guaranteed that all seedlings had the best chance of thriving under controlled conditions.

### 2.2 Experimental Treatments

The experiment involved three different treatment applications: garlic extract, *B. subtilis* , and *Ralstonia solanacearum* or phosphate buffer saline (PBS) inoculation. Garlic cloves were purchased at Johannesburg fresh produce market. After removing the cloves from the skin (husks/peel), each clove was manually peeled. Aqueous garlic extraction was prepared using a kitchen blender according to Ali et al. (2022). About 250 g of crushed garlic paste was mixed with 500 mL of double distilled water in a beaker to obtain a homogenous mixture through stirring. The macerated biomass was kept for 4 days at ambient temperature for the exudation of bio‐chemicals. The biomass was later filtered with muslin cloth to remove any impurities and stored as a stock solution at ambient temperature prior to use.

Pure strain of *Bacillus subtilis* subsp. *spizizenii ATCC 6633* was obtained from Anatech Analytical Technology in Johannesburg, South Africa. The *B. subtilis* isolate was cultured in lysogeny broth at 28°C in the dark for 48 h, and the plate count method was used to estimate cell populations as colony‐forming units per milliliter (CFU mL^−1^), following the procedure described by (Ogugua et al. 2016). To prepare the *B. subtilis spizizeni* medium, 5 g of Triton, 10 g of salt, and 5 g of yeast extract were weighed and dissolved in 1 L of distilled water. For subculturing, the liquid *B. subtilis* culture was gently poured into the prepared liquid medium, mixed thoroughly to ensure even distribution, and placed in a shaker at 30°C for 24 h. After subculturing, the liquid culture was transferred into 50 mL tubes and centrifuged at 3050 rpm for 30 min to separate the bacterial cells. The resulting bacterial pellet was resuspended in distilled water and transferred into clean tubes to ensure purity. The spore count was approximately 10^12^ CFU mL^−1^. Finally, a 3 mL syringe was used to inject the prepared *B. subtilis* suspension into the root zone of the 6‐week‐old tomato plants, ensuring direct interaction between the beneficial bacteria and the plant roots.

Phosphate Buffer Saline (PBS) was prepared according to the manufacturer's guidelines (1 PBS tablet per 200 mL of water) to ensure consistency and accuracy. For larger volumes, 25 tablets were dissolved in 5 L of distilled water, maintaining the same concentration. The solution was thoroughly mixed until the tablets were completely dissolved, resulting in a clear and homogeneous buffer. PBS was administered to the root zone using a 3 mL syringe to ensure direct contact with the plants. This solution served as a mock treatment for all non‐diseased plants that did not receive *R. solanacearum* but was not considered an actual treatment.

*Ralstonia solanacearum* cultures were prepared by isolating a single colony using a sterile loop and transferring it onto solidified nutrient agar. The medium was prepared by dissolving 28 g of nutrient agar powder in 1 L of distilled water, autoclaving at 121°C for 15 min, and pouring it into sterile Petri dishes to solidify. The inoculated plates were sealed and incubated at 25°C–30°C for 48 h. Subculturing was done by transferring a colony into liquid agar medium, mixing evenly, and incubating in a shaker at 30°C for 24 h. For bacterial separation, the liquid culture was centrifuged at 3050 rpm for 30 min, and the pellet was resuspended in sterile water. The bacterial concentration was determined using a spectrophotometer, adjusting the culture to an optical density (OD) of 600 nm, corresponding to approximately 1 × 10^8^ CFU/mL, based on the McFarland standard.

These treatments were applied to evaluate their effects on tomato plant growth and resistance to bacterial wilt. The treatments consisted of *R. solanacearum* , garlic extract, *B. subtilis* , a combination of both, control, garlic extract control, *B. subtilis* control, and combination control.

### 2.3 Experimental Design and Layout

The experimental procedure employed a randomized complete block design (RCBD) involving 72 potted tomato plants grown under controlled greenhouse conditions. The plants were divided into two groups: a diseased group (36 pots inoculated with *Ralstonia solanacearum* ) and a non‐diseased control group (36 pots mock‐inoculated with phosphate‐buffered saline). Each group received four treatments with nine biological replicates per treatment: *R. solanacearum* alone, garlic extract, *Bacillus subtilis* strain, and a combination of garlic extract and *B. subtilis* . To ensure effective delivery of the pathogen and biocontrol agents, treatments were applied using leaf infiltration—where solutions were introduced into the abaxial leaf surface using a sterile needleless syringe—and root drenching, where suspensions were poured directly into the soil near the root zone adapted from Sun et al. (2023). The tomato plants were 10 weeks old at the time of inoculation. Plants were monitored daily for symptom development, and morpho‐physiological parameters such as wilting index, plant height, leaf number, and biomass were recorded at regular intervals until the termination of the experiment.

### 2.4 Metabolite Extraction Procedure

Tomato leaves were collected from all treatments at 14 days post treatment. The experimental plants consisted of *R. solanacearum* , garlic extract, *B. subtilis* , combination, control, garlic extract control, *B. subtilis* control, and combination control. The collected leaves were cleaned to remove any debris, quickly placed in liquid nitrogen, and then immediately stored at −80°C until further analysis. The frozen leaf samples were then pulverized into a fine powder. For extraction, 50 mg of the powdered material was subjected to the methanol extraction method, using 1.5 mL of 80% ice‐cold LC/MS‐grade methanol (HPLC grade, Minema Chemicals). The samples were vortexed for 30 s, sonicated for 2 h, and centrifuged for 5 min at 1485 rpm (Thermo Fisher, Johannesburg, South Africa). The resulting supernatant was filtered through 0.22 μm nylon filters into glass vials containing 500 μL inserts (Agela Technologies, Tianjin, China). Three biological replicates per treatment group were prepared, and the extracts were stored at 4°C prior to analysis.

### 2.5 Liquid Chromatography‐Quadrupole Time of Flight Mass Spectrometry (LC‐QTOF MS) Analysis

Tomato leaf samples from all treatments were analyzed using a liquid chromatography quadrupole time‐of‐flight tandem mass spectrometry instrument (LCMS‐9030 qTOF, Shimadzu Corporation, Kyoto, Japan) to quantify metabolites at 14 days post infection. Chromatographic separation was performed using a Shim‐pack Velox C18 column (100 × 2.1 mm, 2.7 μm particle size) maintained at 55°C. A 3 μL injection volume was used with a binary solvent system comprising 0.1% formic acid in Milli‐Q water (HPLC grade, Merck, Darmstadt, Germany) and methanol (UHPLC grade, Romil Ltd., Cambridge, UK) with 0.1% formic acid. Gradient elution was performed at a flow rate of 0.45 mL/min over a 13‐min runtime, with the following conditions: 10% B for 3 min, gradual increase to 60% B over 3 min, increase to 90% B over 3 min, held for 1 min, and re‐equilibration to 10% B within 1 min, held constant for another 1 min. The qTOF high‐resolution mass spectrometer operated in negative electrospray ionization mode and data‐dependent acquisition. Parameters were optimized following Makhumbila et al. (2023), with interface voltage set at −3.0 kV, interface temperature at 300°C, drying gas flow at 3 L/min, detector voltage at 1.8 kV, flight tube temperature at 42°C, heat block temperature at 400°C, and desolvation line temperature at 280°C.

## 3 Metabolic Data Analysis

### 3.1 Data Pre‐Processing

Data pre‐processing was performed using XCMS online, accessed on 11 November 2024 at 08:35:30 (http://xcmsonline.scripps.edu/) with UPLC‐qTOF parameters set for the centWave feature detection method. A maximum m/z deviation of 15 ppm was allowed, with a signal‐to‐noise threshold of 7, and peak intensity prefilters set at 3 and 700, respectively. Retention time correction was carried out using the obiwarp method with a profStep of 0.5. Alignment was performed with a minimum fraction threshold of 0.5 across all samples and an m/z width of 0.025 for peak grouping. The Kruskal–Wallis non‐parametric test was applied for statistical analysis. The pairwise comparison between garlic treatment and its control yielded a 2.325 feature matrix, *B. subtilis* treatment and its control resulted in a 2.292 feature matrix, while the combined treatments of garlic and *B. subtilis* produced a 2.123 metabolites. The feature matrix from all three treatments was then transferred to SIMCA (Soft Independent Modeling of Class Analogy) version 17.0 software (Sartorius, South Africa). Initially, an unsupervised principal component analysis (PCA) was performed to visualize overall data structure and detect any outliers. Subsequently, a supervised orthogonal projection to latent structures discriminant analysis (OPLS‐DA) was generated to view sample clustering. An S‐plot was generated to identify the metabolites that most significantly contributed to the discrimination between two groups, highlighting those with high covariance and correlation. Partial least‐squares discriminant analysis (PLS‐DA) was also created in comparisons among groups more than 1. Following this, variable importance in projection (VIP, supervised) was generated from PLS‐DA.

### 3.2 Metabolite Annotation, Relative Quantification, and Pathway Analysis

Raw files (mzML) for all treatments were uploaded to the Global Natural Products Social (GNPS) molecular networking platform for spectral analysis and metabolite identification using Winscp. Spectra data were matched against multiple reference libraries, including GNPS, Chemical Entities of Biological Interest (ChEBI), Human Metabolome Database (HMDB), DrugBank, Food Database (DRUGBANK), Food Database (FooDB), and SuperNatural II (SUPNAT). Additional validation was performed using compound databases such as the Kyoto Encyclopedia of Genes and Genomes (KEGG), Metabolomics Database KNApSAcK, Chemical Database (ChemSpider), Compound Database (PubChem), and the Dictionary of Natural Products, using peak mass and isomeric SMILES for comparison. Metabolite annotations were further verified through literature searches to ensure accuracy. The identified metabolites were analyzed for their relative concentrations, and pathway enrichment was assessed using a hypergeometric test. Enrichment analysis was conducted using KEGG metabolite pathways for *Arabidopsis thaliana* in MetaboAnalyst v6.0.

## 4 Results

### 4.1 Multivariate Data Analysis

**FIGURE 1:**
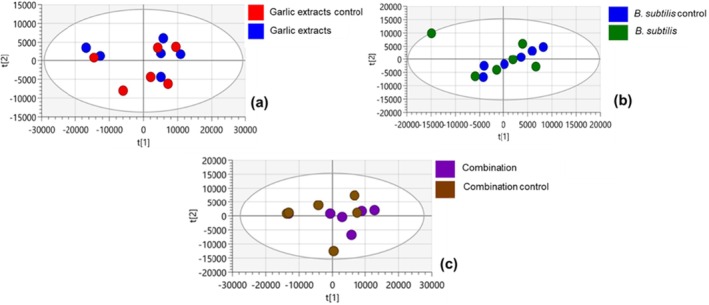
PCA score plots illustrating the overall metabolic differences across the three different treatments and their respective controls: Garlic extracts (a), *B. subtilis* (b), and combined treatment of garlic extract and *B. subtilis* (c).

To identify patterns in the analyzed data, PCA score plot was used where the original variables were transformed into principal components, which highlighted the most important features. Principal component analysis in Figure 1 illustrates the clustering patterns between infected and uninfected tomato plants, highlighting metabolic shifts associated with bacterial wilt infection and treatment effects at the time of experiment termination. The R^2^X(cum) values indicate the proportion of total variance explained by the PCA model, while Q^2^(cum) reflects the model's predictive ability. Garlic extract treatment when compared with control (Figure 1a), the 3‐component PCA model resulted in principal component (PC) 1 of 52.2% and PC2 of 11.5%, which has a total variation of 63.7%. The model showed good fit with R^2^(cum) = 0.733 and predictive ability Q^2^(cum) = 0.0448. Limited or weak separation of treatment groups was observed in the PCA which could mean there was no clear differentiation in the metabolic profiles between the garlic extract treatment and the control. There was no distinct grouping in the *B. subtilis* treatment (Figure 1b), where PCA model resulted in PC1 of 30.5% and PC2 of 8.4%, which resulted in total variation of 38.9%. The 3‐component model showed a good fit with R^2^(cum) = 0.604, and the predictive ability Q^2^(cum) = 0.0186. Figure 1c on the combined treatment of garlic extract and *B. subtilis* also showed no distinct clustering. The 3‐component PCA model resulted in PC1 of 49.3% and PC2 of 15.3%, which resulted in total variation of 64.6%. The model showed good fit with R^2^(cum) = 0.776, and predictive ability Q^2^(cum) = 0.446.

**FIGURE 2:**
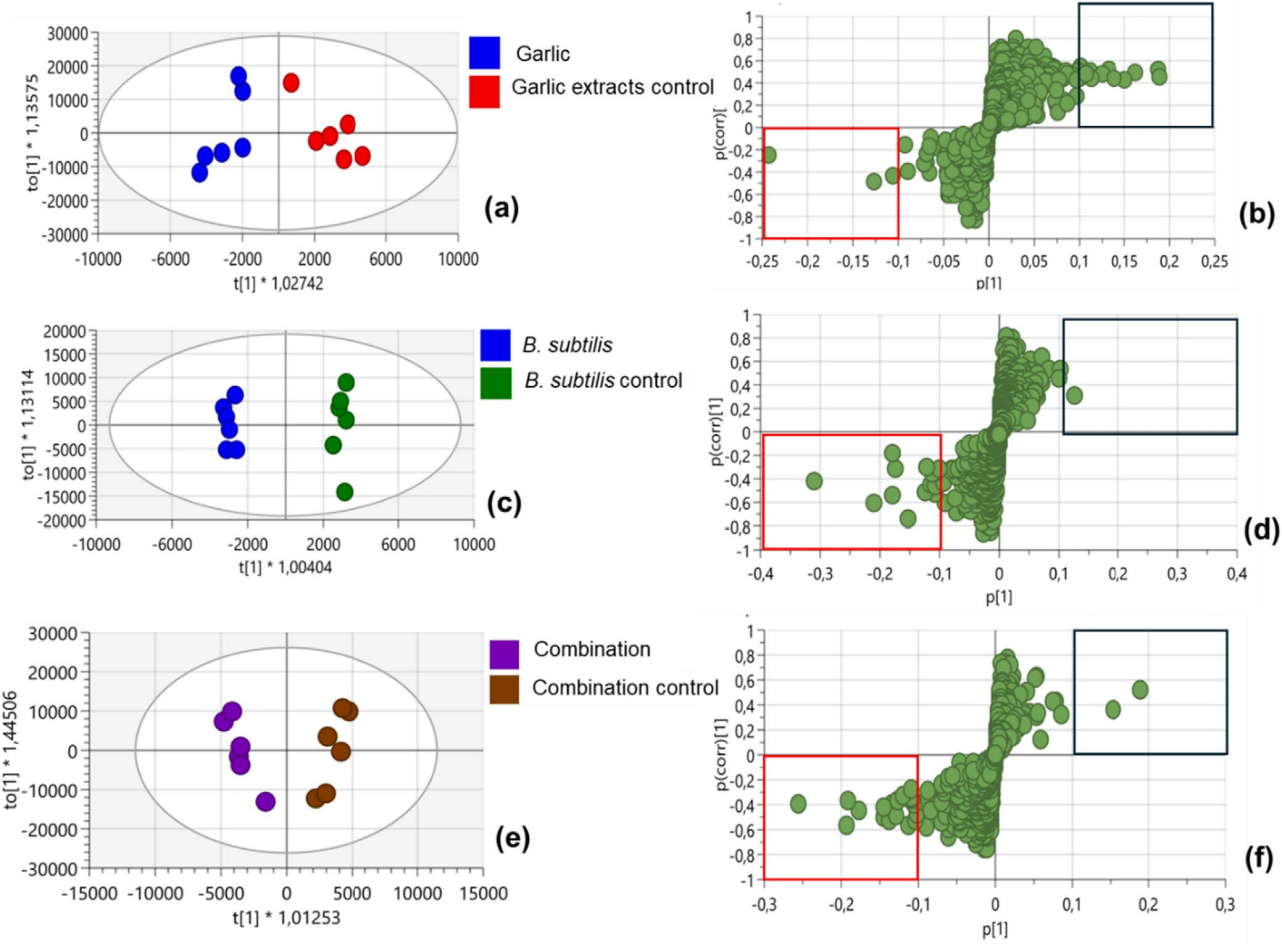
Computed OPLS‐DA and S‐plot loadings score plots showing the overall metabolic differences across treatments and their respective controls. The loadings S‐plot shows the up and down regulation in 0.1 threshold. The black highlighted box on the right shows the up‐regulated metabolites while the red highlighted box on the left shows the down‐regulated in the selected threshold. OPLS‐DA score plot differentiating the metabolomic profiles of diseased tomato plants treated with garlic extract (blue) and its corresponding control (red) (a), and loadings S‐plot showing up‐ and down‐regulated metabolites from garlic treatment (b); OPLS‐DA score plot showing sample clustering of tomato diseased plants treated with *B. subtilis* (blue) and that of *B. subtilis* control plants (green) (c), and loadings S‐plot showing up‐ and down‐regulated metabolites from *B. subtilis* treatment (d); OPLS‐DA plot distinguishing the combined treatment of garlic and *B. subtilis* (purple) compared to the combined control (brown) (e), and (f) the corresponding loadings S‐plot displaying the up‐ and down‐regulated metabolites from the combined treatment.

To further understand the responses and to identify the significant metabolites responsible, OPLS‐DA score plots were generated (Figure 2a,c,e). S‐plot loadings were generated for each treatment from OPLS‐DA (Figure 2b,d,f). In the comparison between garlic extract and its control, OPLS‐DA showed a clear separation showing differentiation in the metabolic profiles (Figure 2a). The 1 + 3 + 0 model component showed a good fit with R^2^(cum) = 0.76, R^2^Y(cum) = 0.867, and predictive ability Q^2^(cum) of −0.48. The score plot contributed to a total variation of 58% in the PC1 and PC2. The generated S‐plot from the garlic treatment (Figure 2b) indicated 10 metabolites that were significantly up‐regulated, while 3 metabolites were down‐regulated. In the comparison between *B. subtilis* and its control, there was clear separation between treatment and control. The OPLS‐DA 1 + 3 + 0 model showed fit with R^2^(cum) = 0.59, R^2^Y(cum) = 0.993, and predictive ability Q^2^(cum) = −0.41. The OPLS‐DA plot yielded a total variation of 34%. In the 0.1 threshold, only 1 metabolite was up‐regulated, whereas 11 were down‐regulated (Figure 2d). There was also clear clustering of groups between combined garlic and *B. subtilis* treatment when compared to its control. The 1 + 3 + 0 model (Figure 2e) showed good fit with an R^2^(cum) = 0.74, R^2^Y(cum) = 0.934, and a predictive ability Q^2^(cum) = 0.478. The PC1 and PC2 showed a total variation of 53%. Only 2 metabolites were up‐regulated, while 12 metabolites were down‐regulated, as shown in the S‐plot loading score plot.

### 4.2 Metabolite Annotation and Comparative Analysis

Metabolites were annotated based on pairwise comparison between treatments and their respective controls from loadings S‐plot showing high significance. A total of 40 metabolites were identified, with 31 being positively annotated (Table 1). Among the different classes, phenolic compounds were the most abundant, followed by flavonoids. Other classes included organic acids, isoprenoids, cyanogenic glycosides, ketones, saponins, lipids, and glucosinolates. Only two metabolites showed a log_2_fold change greater than 1 under *B. subtilis* treatment. The most pronounced change was observed in quinic acid, with a log_2_fold increase of 1.30, followed by 3‐O‐Caffeoyl‐4‐O‐sinapoylquinic acid, which exhibited a log_2_fold change of 1.07 under the same treatment.

**TABLE 1:** Differentially accumulated metabolites in tomato plants infected with *R. solanacearum* across different treatments.

Metabolites	Experimental m/z	Molecular formula	Class	Rt (min)	Treatments					
					Garlic		*B. subtilis*		Combined treatment	
					Log_2_fold	*p*	Log_2_fold	*p*	Log_2_fold	*p*
Caffeoyl glucaric acid	371.062	C_15_H_16_O_11_	Phenolic acid	1.89	0.68	0.68	−0.30	0.30	−2.48	0.10
Chlorogenic acid	353.087	C_16_H_18_O_9_	Phenolic acid	2.51	0.39	0.49	−0.09	0.75	0.38	0.52
Neolinustatin	423.195	C_17_H_29_NO_11_	Cyanogenic glycoside	10.72	−0.45	0.44	0.01	0.99	—	—
3‐O‐Caffeoyl‐4‐O‐sinapoylquinic acid	559.283	C_27_H_28_O_13_	Phenolic acid	7.09	1.07	0.14	—	—	−0.67	0.44
Trihydroxyferuloyl spermidine	721.199	C_37_H_43_N_3_O_12_	Phenolic acid	6.79	0.74	0.17	—	—	−0.63	0.32
Rutin	609.154	C_27_H_30_O_16_	Flavonoid	4.19	−0.25	0.47	—	—	−0.42	0.13
Kaempferol 3‐O‐glucoside	447.371	C_21_H_20_O_11_	Flavonoid	9.88	−0.23	0.69	—	—	−0.76	0.22
Baicalin	445.335	C_21_H_18_O_11_	Flavonoid	9.45	−0.17	0.77	—	—	−0.96	0.14
Quinic acid	191.019	C_7_H_12_O_6_	Organooxygen compounds	0.91	0.04	0.93	1.30	0.10	−0.24	0.50
Octaprenyl diphosphate	722.369	C_40_H_68_O_7_P_2_	Isoprenoid	6.79	0.80	0.15	—	—	—	—
1,14‐Bis(dihydrocaffeoyl)spermine	531.078	C_28_H_42_N_4_O_6_	Phenolic acid	6.47	0.86	0.18	—	—	—	—
Ferulic acid glycoside	355.264	C_16_H_20_O_9_	Phenolic acid	2.84	0.24	0.69	—	—	—	—
p‐coumaroyltriacetate	289.348	C_15_H_13_O_6_	Phenolic acid	3.35	0.09	0.89	—	—	—	—
2‐Cyclohexen‐1‐one	373.140	C_19_H_32_O_7_	Ketone	1.86	—	—	0.63	0.13	—	—
5‐O‐(E)‐Caffeoylgalactaric acid	743.167	C_15_H_1_6O_11_	Phenolic acid	1.76	—	—	0.42	0.20	—	—
Juniperoside III	311.068	C_15_H_20_O_7_	Saponin	8.46	—	—	0.83	0.25	—	—
12‐dienoate	744.990	C_40_H_73_O_10_P	Lipid	1.89	—	—	—	—	−2.48	0.10
3‐butenylglucosinolate	372.065	C_11_H_18_NO_9_S_2_	Glucosinolate	1.86	—	—	—	—	−0.9	0.17
Sinapoylglycoside	385.194	C_17_H_22_O_10_	Phenolic acid	3.64	—	—	—	—	−0.56	0.36

Abbreviations: —, denotes not detected; m/z, mass per charge; Rt, retention time.

Other compounds had log_2_fold changes below 1, with ranges from 0.1 to 0.86. The down‐regulated metabolites had decreasing log_2_fold change (which is negatively correlated). Caffeoyl glucaric acid, chlorogenic acid, and quinic acid were the only three metabolites that were detected in all three treatments in the loadings S‐plot. Other metabolites were only up‐regulated in some treatments while down‐regulated or not present in other treatments. For example, quinic acid was up‐regulated under garlic and *B. subtilis* treatment, showing the highest log_2_fold change of 1.30. However, it was down‐regulated in the combined treatment, suggesting that its expression may be influenced by the interaction between treatments. Additionally, compounds such as kaempferol 3‐O‐glucoside and baicalin were down‐regulated in both the garlic and combined treatments, while none of these three metabolites—including quinic acid—were detected under the *B. subtilis* treatment, indicating a complex and treatment‐specific metabolic response. The down‐regulation in the combined treatment could be influenced by their effect in the garlic treatment.

**FIGURE 3:**
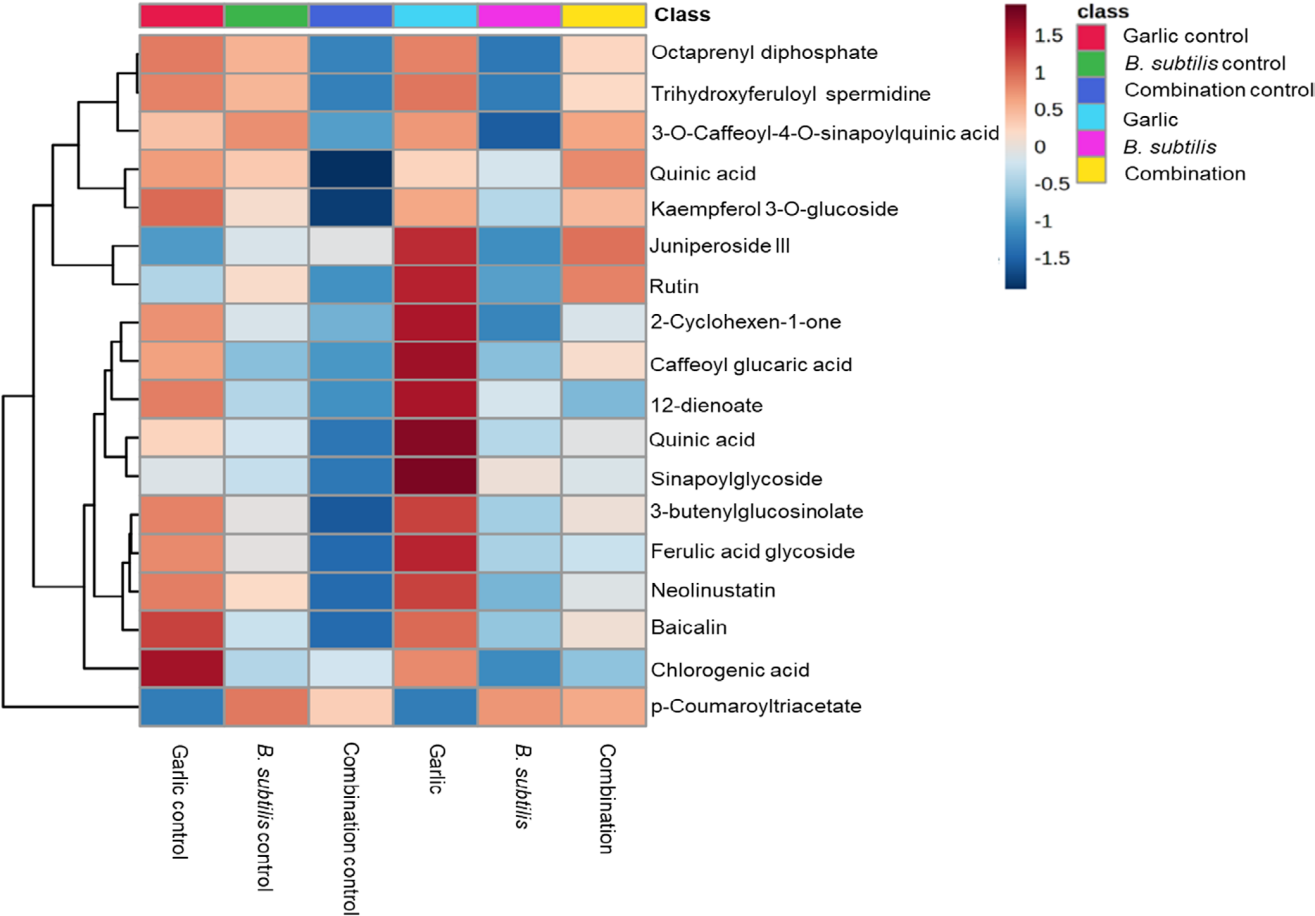
Distribution of annotated metabolites among three treatments according to class. Samples are projected in columns with the metabolites in rows. Color coding indicates abundance (maroon, high abundance; blue, low abundance). The classes represent treatments with garlic (skyblue), garlic extract control (red), *B. subtilis* (purple), *B subtilis* control (green), combined treatment of garlic and *B. subtilis* (yellow) and combined treatment control (blue).

The metabolic shifts from Table 1 were further visualized in Figure 3, which presents a heatmap illustrating the differential accumulation of metabolites across three different treatments. Most metabolites in the garlic treatment were high in abundance as compared to their control, and this could mean that under treatment, the garlic extract induced the up‐regulation of those metabolites. Chlorogenic acid showed high abundance in the garlic control only and showed increasing levels in the plants treated with garlic extract. This metabolite showed low abundance in other treatments, although in Table 1, this metabolite was up‐regulated in combined treatments, but the low abundance in the heatmap agrees with the decreasing log_2_fold changes. Most metabolites under treatment with *B. subtilis* were low in abundance, while in the control, the regulation of those metabolites was at moderate levels. This could mean that the use of this biocontrol agent did not induce the up‐regulation of the key metabolites. Under combined treatment, most metabolites were low in control, while in the treated plants, there was some level of induction as most metabolites were regulated, showing an increase in abundance.

**FIGURE 4:**
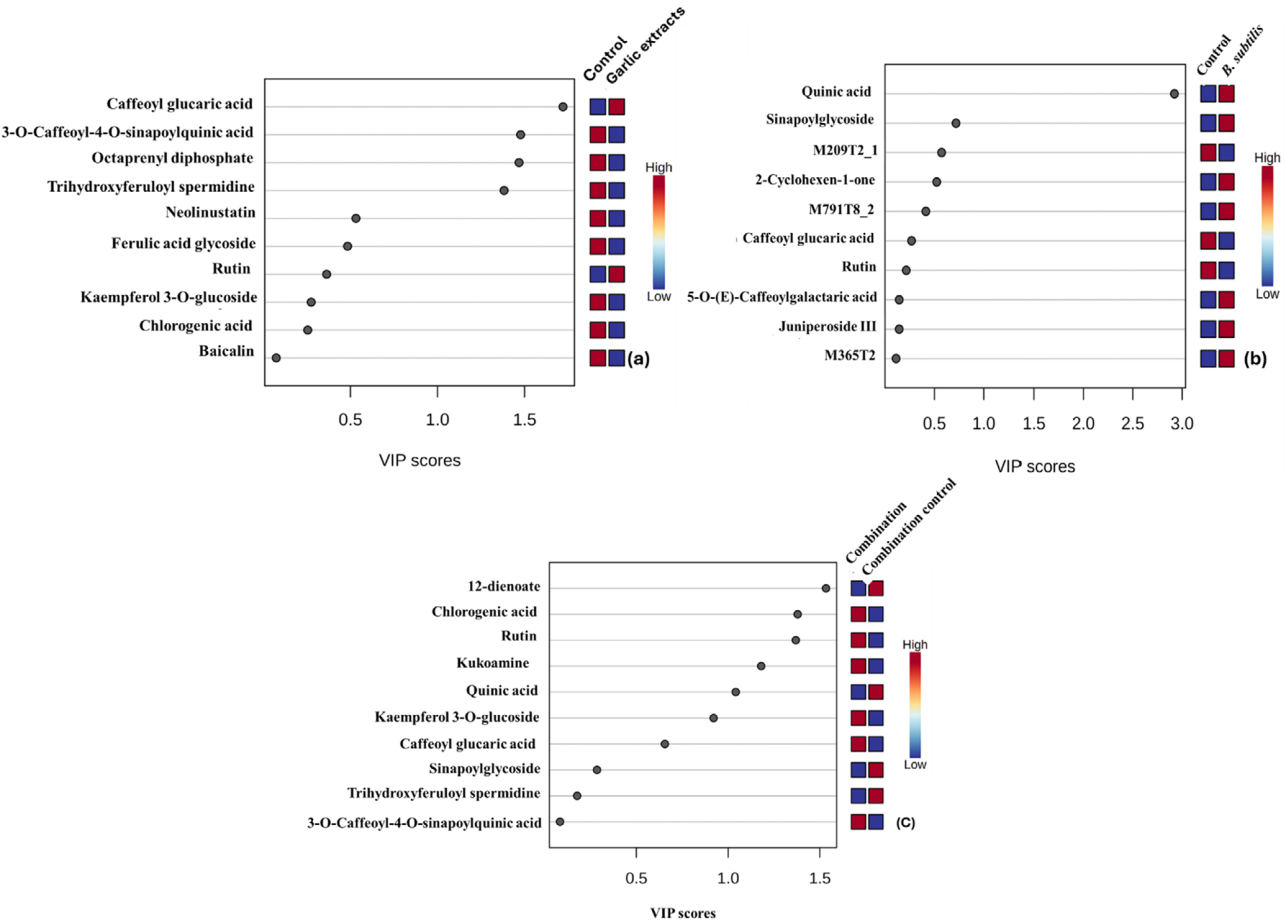
VIP score plots showing metabolites in tomato plants treated with garlic extract, *B. subtilis* , and the combination of garlic extract and *B. subtilis* , with red indicating high abundances and blue low abundances.

The VIP scores (Figure 4) show key metabolites contributing to variation in garlic, *B. subtilis* , and the combined garlic extract and *B. subtilis* treatment, with values ranging from 0.5 to 3.0. Most metabolites showed a high increase in garlic control, while only caffeoyl glucaric acid and rutin were high in garlic treatment (Figure 4a). Metabolites that were significantly high, with the highest VIP score approximately equal to 1.5, were 3‐O‐caffeoyl‐4‐O‐sinapoylquinic acid, octaprenyl diphosphate, and trihydroxyferuloyl spermidine in garlic control. In *B. subtilis* treatment (Figure 4b), most metabolites were significantly high in the garlic treatment as compared to the control, with quinic acid being the only metabolite with the highest VIP score above 3.0. Most metabolites in the combined garlic extract and *B. subtilis* treatment showed high increase levels as compared to those in control (Figure 4c). Among the most influential metabolites, 12‐dienoate, chlorogenic acid, and rutin exhibited high VIP scores above 1.4. Chlorogenic acid and rutin were more abundant in combined treatment, while 12‐dienoate was abundant in control, with the highest VIP score of 1.5.

**FIGURE 5:**
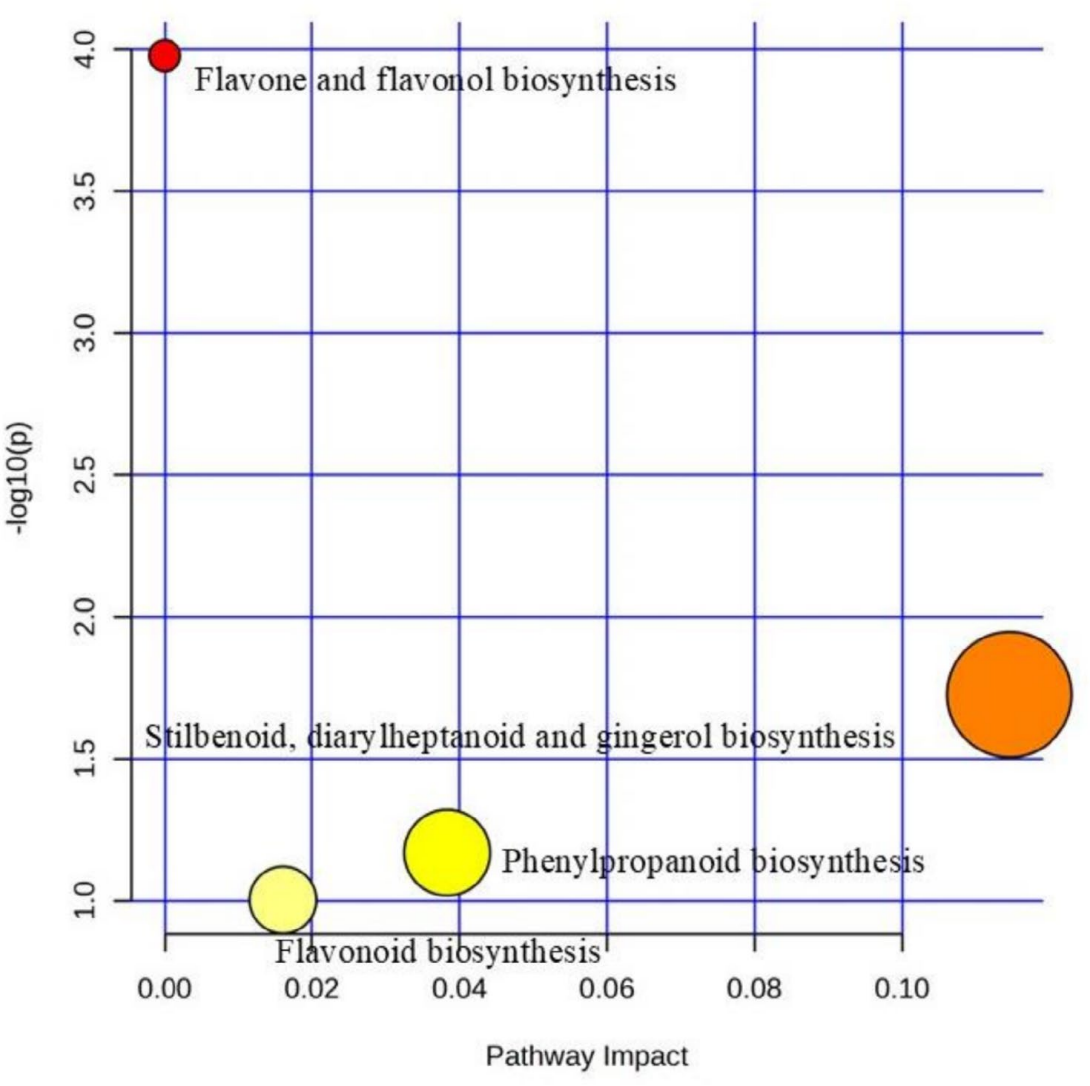
Pathway topology analysis showing the key metabolic pathways impacted under experimental conditions computed from Met Pathway in MetaboAnalyst.

The pathway analysis in Figure 5 and Table 2 shows the major biosynthetic pathways affected, which included flavone and flavonol biosynthesis, stilbenoid, diarylheptanoid, and gingerol biosynthesis, phenylpropanoid biosynthesis, and flavonoid biosynthesis. Flavone and flavonol biosynthesis pathways had the highest log_10_ value; although it was high, the impact of this metabolism was low. The stilbenoid, diarylheptanoid, and gingerol biosynthesis pathways exhibited the highest impact, as indicated by the large node size, and this was followed by phenylpropanoid and lastly the flavonoid biosynthesis. The compounds contributing to these pathways included caffeoyl galactaric acid, ferulic acid glycoside, 12‐dienoate, juniperoside, trihydroxyferuloyl spermidine, and chlorogenic acid.

**TABLE 2:** Pathway enrichment analysis of metabolites in tomato plants in response to *R. solanacearum* infection and biocontrol treatments.

Pathway name	*p*	−log(*p*)	Holm p	FDR	Impact
Flavone and flavonol biosynthesis	1.05364	3.9773	0.0097982	0.0097982	0.0
Stilbenoid, diarylheptanoid and gingerol biosynthesis	0.018776	1.7264	1.0	0.87306	0.11458
Phenylpropanoid biosynthesis	0.067692	1.1695	1.0	1.0	0.03835
Flavonoid biosynthesis	0.099382	1.0027	1.0	1.0	0.0161

## 5 Discussion

Bacterial wilt disease imposes a severe constraint on the yield and sustainability of tomato production globally. The pathogen colonizes the xylem vessels and disrupts water and nutrient transport in infected plants, severely affecting crop growth and productivity (Boakye‐Mensah 2020; Ijaz et al. 2024). To mitigate these impacts, enhancing plant defense mechanisms against bacterial pathogens is crucial for improving crop resilience and sustaining productivity. Metabolomic analysis provides valuable insights into these defense responses by identifying key biochemical changes in tomato plants infected with bacterial wilt (Singh et al. 2023). This approach helps uncover potential biomarkers and defense‐related pathways, contributing to a better understanding of disease resistance and plant health.

The current study investigated the ability of garlic extract and *B. subtilis* to promote tomato growth under *R. solanacearum* infection, focusing on the biochemical responses involved in plant defense. Garlic extract is well documented for its antimicrobial properties and its potential to influence plant metabolism by activating defense‐related pathways (Ali et al. 2021; Ojo et al. 2021), while *B. subtilis* is extensively documented for its ability to promote plant growth and induce systemic resistance against pathogens, primarily through hormonal signaling rather than direct antimicrobial activity (Dimkić et al. 2022). The profiling of infected tomato plants under treatment with garlic revealed key metabolites, including chlorogenic acid, 3‐O‐caffeoylcaffeoyl‐4‐O‐sinapoylquinic acid, caffeoyl glucaric acid, and ferulic acid glycoside (Table 1; Figure 3). According to Kumar et al. (2020), phenolic compounds serve as a primary pathway for the production of antioxidants and antimicrobial agents which are involved in plant defense against diseases. In addition, Zeiss et al. (2019) also noted that these compounds contribute to the formation of structural barriers which can boost pathogen resistance in crops like tomato. Phenolic acids have been reported to enhance pathogen resistance, as demonstrated in tomatoes infected with *Cryptococcus laurentii* (Tang et al. 2022).

The up‐regulation of chlorogenic acid observed in this study—even in the control plants treated with garlic extract—aligns with the findings of Yadav et al. (2020), who reported that chlorogenic acid contributes to strengthening the cell walls of *Capsicum annuum* (chili peppers), thereby limiting pathogen entry. As a key intermediate in the phenylpropanoid biosynthesis pathway, chlorogenic acid plays a crucial role in plant defense by contributing to the formation of lignin and other antimicrobial compounds. Interestingly, although this metabolite was also up‐regulated in the combined garlic extract and *B. subtilis* treatment, its concentration showed a declining trend, and it was down‐regulated under *B. subtilis* treatment alone. This suggests that its regulation may be primarily influenced by the garlic extract, highlighting the extract's potential role in activating phenylpropanoid‐mediated defense responses.

The phenolic compound caffeoyl glucaric acid was found to be up‐regulated in garlic treatment but was down‐regulated in both *B. subtilis* as well as in the combined treatment of garlic extract and *B. subtilis* , as shown in Figure 3. However, this metabolite showed increased abundance in the combined treatment under VIP analysis (Figure 4), despite having a VIP score below 1. According to Padró et al. (2021), caffeoyl glucaric acid exhibits a strong antioxidant property, playing a role in strengthening plant cell walls and scavenging reactive oxygen species (ROS). Its abundance in the combined treatment suggests its important role in eradicating the ROS, although it does not hold high discriminatory power when the garlic is combined with the *B. subtilis* treatment. Additionally, Yang et al. (2024) demonstrated that caffeoyl glucaric acid contributes to lignin biosynthesis and fortifies cell walls in response to *Rhizoctonia solani* infection in potatoes. Djellout et al. (2020) reported that *Bacillus* and *Pseudomonas* strains altered phenolic profiles in *Agrobacterium tumefaciens* infected tomatoes, enhancing antioxidant biosynthesis.

Although in this study not many phenolic compounds were annotated and detected at the studied threshold, their up‐regulation under *B. subtilis* may enhance the antioxidant biosynthesis. Other studies have suggested that *B. subtilis* controls bacterial wilt through antimicrobial lipopeptides (Hyakumachi et al. 2013), while *Bacillus amyloliquefaciens* was found to suppress *R. solanacearum* without promoting phenolic accumulation (Raza et al. 2016), supporting the idea that *Bacillus* species enhance defense through non‐phenolic mechanisms. Hernández‐Pacheco et al. (2022) reported that *Physalis ixocarpa* under salinity stress exhibited increased phenolic acids at prolonged exposure, indicating a dynamic metabolic adjustment to stress conditions. The increased levels of phenolic acids in this study may be attributed to their role in activating the phenylpropanoid pathway, which is crucial for plant defense (Zeiss et al. 2019; Alcázar Magaña et al. 2021).

Another major class of metabolites detected in this study is flavonoids, which play significant roles in oxidative stress responses (Shah and Smith 2020). In the current study, most flavonoids were down‐regulated across treatments at the studied threshold. Their reduced abundance may be attributed to the minimal impact of flavone and flavanol biosynthesis metabolism, as shown in Figure 5. This contrasts with previous studies, where garlic‐derived bioactive compounds were shown to stimulate flavonoid biosynthesis in other plant species. A possible explanation for this decline could lie in metabolic prioritization. According to Parvin et al. (2022), plants under biotic stress often up‐regulate phenolic acids while down‐regulating flavonoids, reallocating metabolic resources toward pathways more critical for defense. A similar trend has been observed in *Solanum lycopersicum* , where, under combined abiotic stress, flavanols accumulated more than hydroxycinnamic acids, effectively mitigating oxidative damage due to their potent antioxidant properties (Martinez et al. 2016). This change in flavonoid abundance demonstrates how secondary metabolism is dynamic under stress, with different compound classes being preferred based on the kind and intensity of environmental stressors.

Lipids play essential roles in signaling and defense responses, particularly in systemic resistance mechanisms (Kuźniak and Gajewska 2024). A reduction in 12‐dienoate in the combined treatment of garlic and *B. subtilis* , and no detection in individual garlic and *B. subtilis* treatments on the studied threshold may suggest that while phenylpropanoid metabolism was enhanced, lipid‐based defense mechanisms may have been down‐regulated, possibly due to competition for metabolic resources. This down‐regulation of lipid metabolism has been reported by Woolfson et al. (2023) in a transcriptomic study on *Solanum tuberosum* , which revealed that genes involved in phenolic production, including those in the shikimate pathway and phenylpropanoid metabolism, were up‐regulated early during wound healing, preceding the transcription of genes associated with aliphatic suberin production. Of which this suggests a temporal coordination of metabolic pathways that may contribute to the observed shifts in lipid metabolism. Juniperoside III was also up‐regulated under *B. subtilis* treatment, suggesting a selective regulation of saponin metabolism (Shukla et al. 2022). Neolinustatin, a cyanogenic glycoside, was down‐regulated in garlic treatment but up‐regulated in *B. subtilis* . Although neolinustatin was down‐regulated under garlic treatment, its concentration remained higher than in the *B. subtilis* treatment. This suggests that garlic's influence, possibly through its organosulfur compounds, as proposed by Smith and Yang (2000), may modulate plant metabolism by affecting enzyme activity involved in detoxification, thereby contributing to the observed metabolic shifts.

Tomato plants infected with *R. solanacearum* and treated with *B. subtilis* in this study exhibited a general down‐regulation of metabolites, with some showing low abundance. Sun et al. (2023) reported similar findings, where *R. solanacearum*‐infected tomato plants treated with *B. subtilis* did not significantly affect secondary metabolite production. They also concluded that the plant may prioritize primary metabolism such as energy production and growth over the production of defense‐related secondary metabolites. Moreover, Yendyo et al. (2018) supported these findings by showing that *B. subtilis* competes with *R. solanacearum* for resources, thus limiting pathogen colonization and thereby reducing the plant's need to allocate resources to secondary metabolite production for defense.

## 6 Conclusion

This study highlights the distinct yet complementary roles of garlic crude extract and *Bacillus subtilis* in enhancing tomato resilience against *Ralstonia solanacearum* . Garlic extracts primarily strengthened plant defense through phenylpropanoid‐mediated mechanisms, increasing key phenolic compounds such as chlorogenic acid and caffeoyl glucaric acid, which reinforce structural barriers and mitigate oxidative stress. In contrast, *B. subtilis* likely relied on microbial competition and hormonal regulation rather than inducing major shifts in secondary metabolism. These findings suggest that plant defense involves trade‐offs between structural reinforcement, resource allocation, and physiological adjustments to stress. However, gaps remain in understanding the precise metabolic mechanisms underlying these effects. The limited up‐regulation of key secondary metabolites such as the phenolics suggests that protection may involve signaling‐based priming or microbial interactions rather than direct metabolic shifts. Further research is needed to determine the optimal concentrations of garlic extract for enhanced metabolic responses and to clarify *B. subtilis* 's mode of action. Future studies should assess the synergistic potential of combined treatments under field conditions to optimize disease management strategies. Understanding the genetic and molecular basis of these responses could support the development of improved biocontrol formulations and bacterial wilt‐resistant tomato varieties. Integrating metabolomic insights with transcriptomic and proteomic approaches may further unravel the regulatory networks governing plant defense, contributing to more sustainable and effective disease management in tomato production.

## Funding

The authors have nothing to report.

## Ethics Statement

The authors have nothing to report.

## Consent

The authors have nothing to report.

## Conflicts of Interest

The authors declare no conflicts of interest.

## Data Availability

All the required data is provided in the manuscript.
